# Site-Specific Conditions Change the Response of Bacterial Producers of Soil Structure-Stabilizing Agents Such as Exopolysaccharides and Lipopolysaccharides to Tillage Intensity

**DOI:** 10.3389/fmicb.2020.00568

**Published:** 2020-04-07

**Authors:** Barbara Cania, Gisle Vestergaard, Marjetka Suhadolc, Rok Mihelič, Maike Krauss, Andreas Fliessbach, Paul Mäder, Anna Szumełda, Michael Schloter, Stefanie Schulz

**Affiliations:** ^1^Research Unit Comparative Microbiome Analysis, Helmholtz Zentrum München, Research Center for Environmental Health (GmbH), Neuherberg, Germany; ^2^Department of Health Technology, Section for Bioinformatics, Technical University of Denmark, Lyngby, Denmark; ^3^Chair of Soil and Environmental Science, Department of Agronomy, University of Ljubljana, Ljubljana, Slovenia; ^4^Department of Soil Sciences, Research Institute of Organic Agriculture (FiBL), Frick, Switzerland; ^5^Stanisław Karłowski Foundation, Silnowo, Poland; ^6^Chair of Soil Science, Technical University of Munich, Freising, Germany

**Keywords:** tillage, soil aggregation, exopolysaccharides, lipopolysaccharides, soil microbiome, metagenomics, *wza*

## Abstract

Agro-ecosystems experience huge losses of land every year due to soil erosion induced by poor agricultural practices such as intensive tillage. Erosion can be minimized by the presence of stable soil aggregates, the formation of which can be promoted by bacteria. Some of these microorganisms have the ability to produce exopolysaccharides and lipopolysaccharides that “glue” soil particles together. However, little is known about the influence of tillage intensity on the bacterial potential to produce these polysaccharides, even though more stable soil aggregates are usually observed under less intense tillage. As the effects of tillage intensity on soil aggregate stability may vary between sites, we hypothesized that the response of polysaccharide-producing bacteria to tillage intensity is also determined by site-specific conditions. To investigate this, we performed a high-throughput shotgun sequencing of DNA extracted from conventionally and reduced tilled soils from three tillage system field trials characterized by different soil parameters. While we confirmed that the impact of tillage intensity on soil aggregates is site-specific, we could connect improved aggregate stability with increased absolute abundance of genes involved in the production of exopolysaccharides and lipopolysaccharides. The potential to produce polysaccharides was generally promoted under reduced tillage due to the increased microbial biomass. We also found that the response of most potential producers of polysaccharides to tillage was site-specific, e.g., *Oxalobacteraceae* had higher potential to produce polysaccharides under reduced tillage at one site, and showed the opposite response at another site. However, the response of some potential producers of polysaccharides to tillage did not depend on site characteristics, but rather on their taxonomic affiliation, i.e., all members of *Actinobacteria* that responded to tillage intensity had higher potential for exopolysaccharide and lipopolysaccharide production specifically under reduced tillage. This could be especially crucial for aggregate stability, as polysaccharides produced by different taxa have different “gluing” efficiency. Overall, our data indicate that tillage intensity could affect aggregate stability by both influencing the absolute abundance of genes involved in the production of exopolysaccharides and lipopolysaccharides, as well as by inducing shifts in the community of potential polysaccharide producers. The effects of tillage intensity depend mostly on site-specific conditions.

## Introduction

Worldwide, 75 billion tons of soil are lost every year by erosion of arable lands (ELD Initiative, 2015). Soil erosion mostly occurs due to the degradation of soil structure, which is defined as the arrangement of soil particles into aggregates (Le Bissonnais et al., 1993). A continuous disruption of soil aggregates by agricultural practices such as conventional tillage (CT) may lead to increased soil compaction as well as loss of organic matter and soil biodiversity. As a result, water retention is impaired, anoxic conditions may appear and nutrient cycling slows down. Such soils are more susceptible to erosion induced by water or wind (Holland, 2004). To protect the aggregated soil structure and prevent soil loss, soil conservation techniques such as reduced tillage (RT) are increasingly encouraged (FAO, 2017).

Although many researchers (Jacobs et al., 2009; Mikha et al., 2013; Bartlova et al., 2015; Sheehy et al., 2015; Singh et al., 2016) observed that soil aggregates are better preserved under RT compared with CT, others (Asgari, 2014; Cania et al., 2019a) reported no effect of tillage intensity on soil aggregation. According to Cooper et al. (2016), the strength of the positive effects of RT strongly depends on soil texture. Clay particles have much stronger aggregating properties compared with silt and sand (Weil and Brady, 2017). Therefore, the positive effects of RT should be more emphasized in soils with lower clay content, where maintaining high aggregation is more challenging (Cooper et al., 2016). This is in agreement with studies using clayey soils that revealed no differences in aggregate stability when RT and CT were compared (Asgari, 2014; Cania et al., 2019a). However, soil aggregation is a complex process that is driven by both abiotic and biotic factors (Six et al., 2004), and still little is known about how tillage intensity influences soil biota and their capabilities to trigger aggregate formation. Babin et al. (2019) proposed that the effects of tillage intensity on soil biota and aggregate formation driven by them can be better studied in soils with lower clay content due to their lower buffering capacity. In accordance with this hypothesis, studies on sandy and silty soils showed that improved soil aggregation under less intense tillage corresponded to increased fungal biomass and glomalin production (Beare et al., 1997; Wright et al., 1999; Cookson et al., 2008; Dai et al., 2015; Lu et al., 2018). However, while many researchers investigated the influence of tillage intensity on the aggregating capabilities of fungi, less attention was given to bacteria. In fact, only in a recent study (Cania et al., 2019a), we compared the effects of CT and RT on the bacterial potential for soil aggregation. Here, we investigated the bacterial potential for the production of exopolysaccharides (EPSs) and lipopolysaccharidesof (LPSs), which act as binding agents for soil particles (Six et al., 2004; Costa et al., 2018; Totsche et al., 2018). EPSs are high-molecular-weight sugars exuded by a wide range of taxa (Suresh Kumar et al., 2007), whereas LPSs are complex glycolipids attached to the outer membrane of most Gram-negative bacterial cells (Whitfield and Trent, 2014). Bacteria use these compounds for attachment to soil particles, which promotes the formation of soil aggregates (Jacques, 1996; Sutherland, 2001). According to Lehmann et al. (2017), the bacterial production of adhesive polysaccharides is one of the most crucial biotic mechanisms of soil aggregation. We (Cania et al., 2019a) could show that while the relative abundance of bacteria capable to form EPSs and LPSs was comparable between CT and RT, the community composition of the potential producers of these compounds differed. As the aggregating efficiency of adhesive polysaccharides produced by different taxa varies greatly (Costa et al., 2018), tillage impact on the community composition of EPS and LPS producers could be critical for the stability of agricultural soils.

However, disentangling the link between soil aggregate stability, tillage and soil texture requires the analysis of long-term experiments, as tillage effects build up over time (Stockfisch et al., 1999; Grandy et al., 2006). Therefore, it was our aim to investigate to which extend the response of polysaccharide-producing bacteria to tillage intensity is driven by the differences in soil texture at different long-term experimental sites, and how this is connected to the stability of soil aggregates. As we were interested in the long-term tillage impact, we focused on parameters that change over a long time period such as aggregate stability and the structure and genetic potential of soil bacterial communities, as opposed to transient parameters such as gene expression and the contents of EPSs and LPSs in soil (Redmile-Gordon et al., 2020). We expected to see more differences in the relative abundance and community composition of potential EPS and LPS producers between CT and RT in silty and sandy soils compared with clayey soils. We also assumed that the differences in the potential to produce bacterial polysaccharides would be reflected by changes in aggregate stability. To address our research questions, we performed a high-throughput shotgun sequencing of DNA extracted from conventionally and reduced tilled soils from three long-term field trials characterized by different soil textures (clayey, loamy, sandy). We used a targeted bioinformatics pipeline to analyze bacterial communities potentially involved in the production of EPSs and LPSs.

## Materials and Methods

### Sites Description and Sampling

Soil samples were taken from three field trials that differed in soil properties but were comparable in terms of soil management, particularly tillage application (CT and RT). The basic information on the trials, such as the locations, soil types and climatic conditions, is summarized in Table 1.

**TABLE 1:** Trial characteristics.

**Trial**	**Frick**	**Moškanjci**	**Juchowo**
Trial start	2002	1999	2010
Geographic coordinates	47°30′N, 8°01′E	46°03′N, 15°04′E	53°40′N, 16°30′E
Elevation [m a.s.l.]	350	225	160
Soil type	Stagnic Eutric Cambisol	Skeletic Eutric Cambisol	Haplic Arenosol
Soil texture	clayey	loamy	sandy
Climate type	temperate	continental	continental
Mean annual temperature [°C]	8.9	10.6	8.5
Mean annual precipitation [mm]	1000	913	750

At the Frick trial (Switzerland), CT has been based on ploughing with a moldboard plough operating at 15–18 cm depth, while for RT, soil loosening has been performed at a depth of 5–10 cm with a chisel and a skim plough, with occasional non-inversion loosening to 15–20 cm. In both systems, the seedbed preparation has been done using a rotary harrow running at 5 cm depth. The last tillage operations before sampling occurred in September 2016 right after harvesting spelt and before grass-clover was sown. In 2017, five grass-clover harvests were done in April, June, July, September and November. The plots were fertilized with slurry in 2016 in August, and in 2017 in April, June and August at the rates of 45, 40, 25 and 30 m^3^ ha^–1^, respectively, before sowing and after the first, second and third grass-clover harvest.

At the Moškanjci trial (Slovenia), for CT, a moldboard plough operating at 20 cm depth has been used, followed by soil bed preparation with a rotary hoe. For RT, a special machine – 4-row disc harrow with individually suspended discs and a system for varying the working angle (and thus the tilling intensity) – has been applied in one or two passes to till the soil and prepare the seedbed. The depth of RT was up to 10 cm. In both systems, the main soil tillage was done in October 2016, just before winter rye was sown. After the winter rye was harvested in July 2017, a mixture of cover crops was sown for green manure. The fertilization with slurry was applied in March 2017 in the amount of 20 m^3^ ha^–1^.

At the Juchowo trial (Poland), CT has been performed by ploughing up to 30 cm deep with an Ecomat plough, while for RT, soil loosening up to 10 cm deep has been done using a cultivator with goosefeet sweeps. In August 2015, the last tilling occurred after harvesting spelt and before lupine was sown. Slurry was applied in March 2015 at the rate of 16 m^3^ ha^–1^. Biodynamic preparations, consisting of subtle amounts of silica (horn silica 501) or fresh cow manure (horn manure 500) dissolved in a large volume of water, were sprayed at the rates of 12 L ha^–1^, 35 L ha^–1^, and 12 L ha^–1^ in March, April and May, respectively. Kieserit (MgO 25%, S 20%) was applied in May at the rate of 50 kg ha^–1^.

Each trial consisted of four replicated plots per treatment, out of which three were sampled in spring, before any tilling and subsequent sowing started, in 2016 in Juchowo, and in 2018 in Frick and Moškanjci. Approximately 10 cores per plot were taken using soil augers with a diameter of 2.5 cm to a soil depth of 10 cm. Cores from the same plot were homogenized, resulting in 18 samples (3 trials x 2 tillage treatments x 3 plot replicates). After being directly cooled in the field, one part of each homogenized soil sample was stored at 4°C and used for physicochemical measurements, and the other was stored at −20°C for DNA extraction and sequencing.

### Physicochemical Measurements

Stable aggregate fraction (SAF) of the soils was determined using a wet sieving method described by Murer et al. (1993). Field-moist soil samples, 4 g of each, were placed onto 0.25 mm sieves of a sieving apparatus, and immersed in water. After 5 min of wet sieving, the aggregates remaining on the sieves were dried at 105°C and weighed. The aggregates were then destroyed by covering them with 0.1 M sodium pyrophosphate (Na_4_P_2_O_7_) solution for 2 h. The remaining particles >0.25 mm (sand and organic debris) were dried and weighed again. The SAF was calculated as percentage of stable aggregates in a moist sample, after applying a correction for sand particles. Aside from using moist soil without previous fractionation, the technique followed the details according to Murer et al. (1993). Soil texture was determined using a combined sieving and sedimentation approach (ISO 11277 2009). The determination of soil organic carbon (Corg) was based on the Walkley-Black wet digestion procedure (Walkley and Black, 1934). The measurement of dissolved organic carbon (DOC) and nitrogen (DON), as well as Cmic and nitrogen (Nmic) was accomplished by means of the chloroform fumigation-extraction (CFE) method (Brookes et al., 1985; Vance et al., 1987). DOC and DON were measured in unfumigated samples, while Cmic and Nmic were calculated as a difference in extractable carbon and nitrogen between fumigated and unfumigated soils. To calculate the microbial biomass, the difference was then multiplied with the empirical factors kEC and kEN to achieve Cmic (kEC = 0.45) and Nmic (kEN = 0.54) (Joergensen, 1996; Joergensen and Mueller, 1996). The measurement of pH was performed in a 1:2.5 (v/v) suspension of soil in demineralized water (pH in H_2_O) after standing overnight (ISO 10390 2005).

### DNA Extraction, Library Preparation and Sequencing

Metagenomic DNA was extracted from 0.5 g of frozen soil according to the phenol-chloroform based DNA/RNA co-extraction protocol described by Lueders et al. (2004). Sample lysis was performed using CKMix tubes and a Precellys24 homogenizer (Bertin Technologies, France). Extracted DNA was quantified by means of a Qubit 4 Fluorometer and a Qubit dsDNA BR Assay Kit (Thermo Fisher Scientific, United States). The purity was also assessed by measuring the A260 nm/A280 nm and A260 nm/A230 nm ratios using a NanoDrop 1000 spectrophotometer (Thermo Fisher Scientific, United States). After extraction, DNA was stored at −20°C until further processing.

1 μg of DNA per sample was sheared using an E220 Focused-ultrasonicator (Covaris, United States), targeting 500 bp fragments as described in the protocol of the producer. Metagenomic libraries were constructed with 100 ng of the sheared DNA by means of a NEBNext Ultra II DNA Library Prep Kit for Illumina and NEBNext Multiplex Oligos for Illumina (New England Biolabs, United Kingdom). Following the manufacturer’s guideline, the provided adaptor was diluted 10-fold to prevent the occurrence of dimers. Size selection was carried out with Agencourt AMPure XP beads (Beckman Coulter, United States), selecting for libraries with 500–700 bp inserts. The beads were also used for purification of PCR reactions and an additional final purification step to eliminate residual primer dimers (1:0.6 DNA to bead ratio). PCR amplification was performed with 12 cycles.

Library size and concentration were evaluated using a Fragment Analyzer and a DNF-473 Standard Sensitivity NGS Fragment Analysis Kit (Advanced Analytical, United States). Libraries were pooled equimolarily (4 nM), and 17 pM of the mixture was spiked with 1% PhiX. Paired-end sequencing was performed on a MiSeq sequencer using a MiSeq Reagent Kit v3 for 600 cycles (Illumina, United States). As a negative control, a reagent-only sample was processed alongside the biological samples at the DNA extraction and library preparation steps, and included in the sequencing run. Raw sequencing data obtained from the MiSeq is available at the Sequence Read Archive (SRA) under the accession number PRJNA555481.

### Bioinformatical Analysis

The raw sequencing data was filtered according to Vestergaard et al. (2017). Remnant adaptor sequences were removed by means of AdapterRemoval v2.1.7 (Schubert et al., 2016). Using the same program, terminal nucleotides with Phred quality scores lower than 15 were trimmed, and reads shorter than 50 bp were discarded. PhiX contamination was removed by means of DeconSeq v0.4.3 (Schmieder and Edwards, 2011).

Filtered reads were taxonomically classified by aligning against the National Center for Biotechnology Information Non-Redundant (NCBI-NR) protein sequences database (January 2017) using Kaiju v1.4.4 (Menzel et al., 2016) in Greedy mode with five allowed mismatches. 16S rRNA gene sequences were additionally identified using SortMeRNA v2.0 (Kopylova et al., 2012) with the SILVA SSU database (release 132).

Subsequent assignment of genes specific for EPS and LPS biosynthesis was performed only for reads classified by Kaiju as bacterial. Hidden Markov model (HMM) searching combined with blasting against protein sequences obtained from the Kyoto Encyclopedia of Genes and Genomes (KEGG) database (October 2016) was carried out according to Cania et al. (2019a). HMMs were downloaded from the TIGRFAMs (version 15) (Haft et al., 2013) and Pfam (version 30) (Finn et al., 2016) databases. Open-reading frames were predicted using FragGeneScan v1.19 (Rho et al., 2010), and then scanned using HMMER v3.1b2 (Mistry et al., 2013). Hits with a maximum *E*-value of 1 × 10^–5^ were mapped to KEGG Orthology (KO) numbers. KO numbers were assigned to the reads for which the best 25 blast results were matching. Blasting was performed by means of Diamond v0.8.38 (Buchfink et al., 2015) with more-sensitive parameters. Genes *epsA* and *epsG* had only a few reads annotated to them (7 and 4, respectively), mostly in single replicates. Therefore, they were excluded from the analysis, as a higher sequencing depth would be required to study them. The list of HMMs and KO numbers used in this study is presented in Table 2.

**TABLE 2:** Proteins related to exo- and lipopolysaccharide production with corresponding KO numbers, HMM IDs and genes.

**Protein**	**KO number**	**HMM ID**	**Gene**
polysaccharide export outer membrane protein Wza	K01991	PF02563	*wza*
colanic acid biosynthesis acetyltransferase WcaB	K03819	TIGR04016	*wcaB*
colanic acid biosynthesis acetyltransferase WcaF	K03818	TIGR04008	*wcaF*
colanic acid/amylovoran biosynthesis pyruvyl transferase WcaK/AmsJ	K16710	TIGR04006	*wcaK*/*amsJ*
capsular polysaccharide export system permease KpsE	K10107	TIGR01010	*kpsE*
alginate export outer membrane protein AlgE	K16081	PF13372	*algE*
alginate biosynthesis acetyltransferase AlgJ	K19295	PF16822	*algJ*
levansucrase SacB	K00692	PF02435	*sacB*
lipopolysaccharide transport system ATP-binding protein Wzt	K09691	PF14524	*wzt*
LptBFGC lipopolysaccharide export complex inner membrane protein LptC	K11719	TIGR04409, PF06835	*lptC*
LptBFGC lipopolysaccharide export complex permease LptF	K07091	TIGR04407	*lptF*
LptBFGC lipopolysaccharide export complex permease LptG	K11720	TIGR04408, PF03739	*lptG*

### Statistical Analysis and Data Visualization

Statistical analysis and data visualization were performed using R v3.4.4 (R Core Team, 2016). Sequencing data was analyzed based on relative abundance of reads obtained by dividing the number of reads assigned to a bacterial family or gene, by the total number of bacterial reads per sample. The resultant decimals were subsequently multiplied by 100 in order to convert them into percentages. Absolute gene abundance was additionally estimated according to Zhang et al. (2017) by multiplying the relative gene abundance in percentage with the Cmic value, and dividing by 100. Although Cmic includes both bacterial and fungal carbon, the low and stable values of the Cmic/Nmic ratio indicate that the estimated values are rather precise (Cheng et al., 2013). A robust 2-way independent analysis of variance (ANOVA) was used to detect global effects of trial, tillage intensity and their possible interaction in the whole dataset. Local effects of tillage intensity were detected separately in the datasets from each trial by means of a robust *t*-test. Both statistical tests were based on the median as M-estimator, and used 2000 bootstrap samples. The tests were ran by employing the pbad2way and pb2gen functions from the WRS package (Wilcox and Schönbrodt, 2014). The influence was regarded as significant if the *p*-value was smaller than 5 % (*P* < 0.05). The false discovery rate was controlled in the data derived from the metagenomic datasets with the Benjamini-Hochberg procedure. Effect sizes were calculated in the form of omega squared (ω^2^) and Pearson’s correlation coefficient r as described by Field et al. (2012). The dissimilarity between the samples was explored using principal component analysis (PCA) ordination created with the rda function in the vegan package (Oksanen et al., 2018). For the same purpose, Bray-Curtis dissimilarity matrices were calculated by means of the vegdist function, also from the vegan package. Negative eigenvalues were corrected using the Caillez procedure (Cailliez, 1983). The Bray-Curtis distances were used to create principal coordinate analysis (PCoA) ordinations by means of the pcoa function from the ape package (Paradis et al., 2004). The PCA ordination was generated for the soil properties data, whereas the PCoA ordinations were generated for the sequencing data. For both types of ordination plots, ellipses representing 95 % confidence level were drawn around triplicates with the ellipse package (Murdoch and Chow, 2018).

## Results

### Soil Properties

**FIGURE 1:**
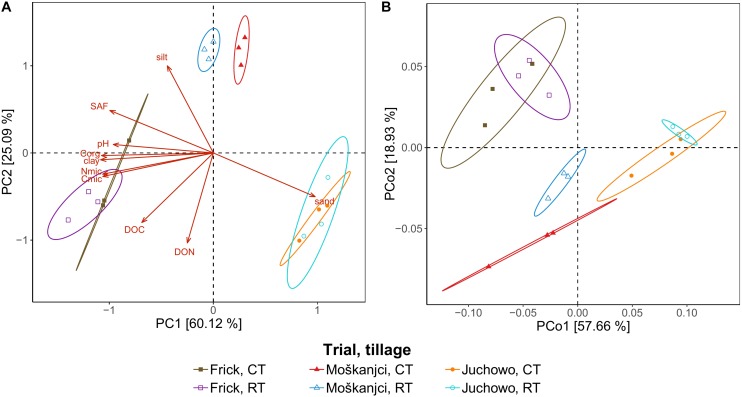
PCA of soil parameters **(A)**, and PCoA based on Bray-Curtis distances depicting taxonomic profiles of bacteria at the family level **(B)**. Ellipses drawn around triplicates represent a 95% confidence level.

Different soil texture in Frick (46.5% clay, 24.5% silt, and 29.0% sand), Moškanjci (17.6% clay, 43.4% silt, and 39.0% sand) and Juchowo (2.4% clay, 6.4% silt, and 91.2% sand) was the primary reason why these trials were included in the current study. However, soil samples of CT and RT from the trials differed also in several other measured physical, chemical and biological parameters (Table 3). The parameters measured as part of the current study included soil texture, SAF, Corg, DOC, DON, pH, Cmic, Nmic, and the Cmic/Nmic ratio. PCA (Figure 1A) of these parameters revealed that the samples were separated primarily based on their place of origin. However, in Moškanjci, soil properties differed also between tillage treatments. Statistical analysis confirmed these observations. Aside from the soil texture data, robust ANOVA detected significant differences in Corg, DOC, DON, Cmic and Nmic values between the trials. Corg was highest in Frick (2.66%), intermediate in Moškanjci (1.59%) and lowest in Juchowo (0.81%). DOC was highest in Frick (68.8 mg/kg), intermediate in Juchowo (42.2 mg/kg) and lowest in Moškanjci (22.5 mg/kg). DON was higher in Frick and Juchowo (12.0 and 10.8 mg/kg, respectively) compared with Moškanjci (3.1 mg/kg). Cmic and Nmic were highest in Frick (1341.9 and 191.3 mg/kg, respectively), intermediate in Moškanjci (342.2 and 53.0 mg/kg, respectively) and lowest in Juchowo (121.5 and 18.5 mg/kg, respectively). Moreover, robust ANOVA identified significant interaction effects between trial and tillage intensity on SAF and pH. Both parameters were highest in Frick (94.3% and 7.2, respectively), intermediate in Moškanjci (69.3% and 6.7, respectively) and lowest in Juchowo (5.5% and 6.3, respectively). Interestingly, SAF and pH were similar under CT and RT in Frick and Juchowo, but higher under RT compared with CT in Moškanjci. Furthermore, robust ANOVA detected significant differences between tillage intensities in Corg, Cmic, and Nmic, with higher values of all of them under RT compared with CT in all three trials. However, the Cmic/Nmic ratios were not significantly influenced by the type of tillage, and did not vary between the sites. The described data is summarized in Table 3.

**TABLE 3:** Soil texture (clay, silt and sand content), stable aggregate fraction (SAF), organic carbon (Corg), dissolved organic carbon (DOC) and nitrogen (DON), pH, microbial biomass carbon (Cmic) and nitrogen (Nmic) as well as the Cmic/Nmic ratio data of the soils under conventional (CT) and reduced (RT) tillage. Influence of trial, tillage and their interaction was determined by a robust 2-way ANOVA.

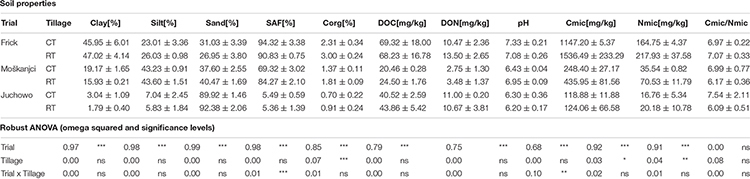

*Effect sizes (ω^2^) and significance levels were calculated based on triplicates (*n* = 3). Significance levels are represented by the amount of stars: 1–*p* < 0.05, 2–*p* < 0.01, 3–*p* < 0.001.*

### Baseline Data of the Shotgun Sequencing

Shotgun sequencing of 18 libraries resulted in 14.91 Gbases of data in total (49.54 million reads with a length of 301 bp each). Following quality control, the metagenomic datasets consisted of 14.73 Gbases (49.53 million reads). The number of filtered reads per sample ranged from 1.6 to 3.7 million, with a mean of 2.8 million. The average length of reads after filtering varied between samples from 292 to 298 bp, with a mean of 297 bp. More details of the raw and filtered sequencing data can be found in Supplementary Table S1. No reads were obtained for the negative control, confirming a lack of contamination during DNA extraction and library preparation.

### Taxonomic Profiling

When all filtered reads were aligned against the NCBI-NR database, 76.8% were assigned to *Bacteria*, 2.9% to *Eukaryota*, 0.6% to *Archaea*, 0.1% to *Viruses* and 19.6% were unclassified. The results of the taxonomic profiling using the NCBI-NR database were supported by SILVA’s assignment of the 16S rRNA gene. Even though only 0.07% of all filtered reads were annotated to the 16S rRNA gene, bacterial communities showed similar distribution patterns regardless of the analytical approach (data not shown). For this reason, as well as because the focus of this study was on EPS and LPS producers of bacterial origin, only bacterial reads identified by means of the NCBI-NR database were analyzed further.

64.2% of the bacterial reads could be assigned at the family level. In total, 385 bacterial families were detected in the datasets, out of which 379 were present under both tillage treatments in all three trials. The remaining six families were very low abundant and altogether accounted for 0.0002% of all bacterial reads. In comparison, the most dominant families – *Bradyrhizobiaceae*, *Streptomycetaceae*, and *Sphingomonadaceae* – comprised 4.6%, 3.1%, and 2.2% of the bacterial reads, respectively. In total, fourteen families each accounted for more than 1% of all bacterial reads, and thus were dominant in the metagenomic datasets (Supplementary Figure S1). These families were all members of the phyla that ranked in the top ten most abundant in our study, led by *Proteobacteria* and *Actinobacteria*, which represented 37.5% and 20.8% of the bacterial reads, respectively (Supplementary Figure S2). Even though almost all of the detected families were present in all samples, their relative abundance differed between the trials or tillage types. As revealed by robust ANOVA, *Bradyrhizobiaceae* were more abundant in Juchowo (6.4%) compared with Moškanjci (3.7%) and Frick (3.4%), whereas *Sphingomonadaceae* were most abundant in Moškanjci (2.6%), intermediate in Juchowo (2.3%) and least in Frick (1.9%). Aside from *Bradyrhizobiaceae* and *Sphingomonadaceae*, another ten dominant families were significantly affected by trial, and three of them were additionally influenced by tillage treatment. In total, 237 families were influenced only by trial, 1 – only by the type of tillage, 4 – by both factors, and 8 – by the interaction of both factors. Interestingly, all the families that were significantly affected by tillage intensity (*Mycobacteriaceae*, *Nocardioidaceae*, *Micromonosporaceae*, *Glycomycetaceae* and *Dermatophilaceae*) were members of *Actinobacteria*. All of them were more abundant under RT compared with CT. The full list of impacted families can be found in Supplementary Table S2; the significance levels and ω^2^ values for the dominant families are listed in Supplementary Table S3. PCoA (Figure 1B) confirmed that the relative abundances of bacterial families were affected primarily by trial, whereas the influence of tillage intensity played only a minor role in shaping the bacterial communities, and was visible mainly in Moškanjci. The results of the PCoA highly resembled those of the PCA of soil properties (Figure 1A), indicating that local conditions strongly affect the composition of bacterial communities and/or vice versa.

### Genes Catalyzing EPS and LPS Biosynthesis

**FIGURE 2:**
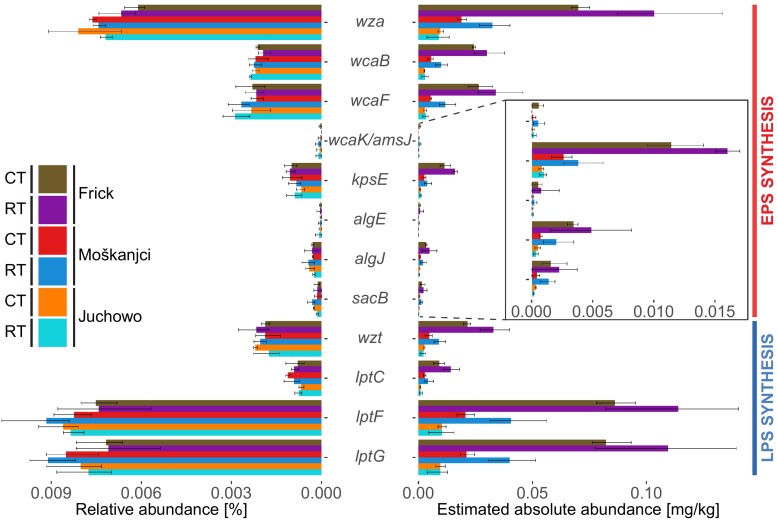
Comparison of the relative and absolute (estimated based on Cmic values) abundances of genes related to the formation of EPSs and LPSs. Error bars show standard deviations.

Genes specific for the synthesis and export of colonic acid, alginate and other EPSs as well as LPSs (Table 2), were identified using an approach combining hidden Markov model (HMM) searches with blasts against sequences derived from the Kyoto Encyclopedia of Genes and Genomes (KEGG) database. In total, the investigated genes comprised 0.033% of all bacterial reads (Figure 2). A rarefaction analysis (Supplementary Figure S3) confirmed that the sequencing depth was sufficient to capture the total diversity of these genes in the analyzed samples. Dominant genes, with a relative abundance between 0.007% and 0.008% of all bacterial reads, were *wza, lptF*, and *lptG*. Genes *wcaB*, *wcaF, kpsE*, *wzt*, and *lptC* were moderately abundant (0.001% – 0.002%). The least abundant (>0.001%) were genes *wcaK*/*amsJ*, *algE*, *algJ*, and *sacB*. Robust ANOVA revealed that only the relative abundance of *wza* differed significantly between samples. This gene was more abundant in Moškanjci and Juchowo than in Frick. The relative abundance of the other investigated genes was not significantly affected by either trial or tillage intensity. The significance levels and ω^2^ values for the investigated genes can be taken from Supplementary Table S4.

Compared with the relative abundance, the absolute abundance of genes related to the formation of EPSs and LPSs, estimated based on the Cmic values, showed higher variability (Figure 2). Robust ANOVA (Supplementary Table S5) indicated a significant impact of trial on most of the investigated genes. Specifically, *wza*, *wcaB*, *wcaF*, *kpsE*, *algJ*, *sacB*, *wzt*, *lptC*, *lptF* and *lptG* were most abundant in Frick, and least in Juchowo. Only *wcaK*/*amsJ* and *algE* were not significantly affected by trial. The estimated absolute abundance of none of the analyzed genes was significantly influenced by the type of tillage. However, robust ANOVA mostly describes differences in the complete dataset, and thus small but significant differences might be missed by this approach. Therefore, to identify site-specific impacts of the different forms of tillage on the estimated absolute abundance of the investigated genes, metagenomic datasets from the different trials were analyzed separately. Robust *t*-test (Supplementary Table S5) revealed three genes that responded to tillage intensity in Frick, eight in Moškanjci and one in Juchowo. In Frick, *wza*, *kpsE* and *wzt* were more abundant under RT compared with CT. Similarly, *wza*, *wcaB*, *wcaF*, *algJ*, *sacB*, *wzt*, *lptF* and *lptG* had higher abundances under RT compared with CT in Moškanjci. In Juchowo, *sacB* was more abundant under CT compared with RT.

### Potential EPS/LPS Producers

The investigated genes were found in 260 bacterial families, including all dominant families (Supplementary Figure S1). Robust ANOVA detected that the potential of the bacterial community to produce EPSs and LPSs was most affected by trial alone and the interaction of tillage and trial rather than by tillage alone. The significance levels and ω^2^ values for the dominant families are listed in Supplementary Table S6, and the full list of impacted families can be found in Supplementary Table S2. The relative abundance of reads related to EPS and LPS synthesis and export differed significantly between trials for five of the dominant families, and for further six among the less abundant members of the community of potential polysaccharide producers. Another six of the less abundant families were affected by the interaction of trial and tillage intensity, but none was influenced by tillage treatment alone.

**FIGURE 3:**
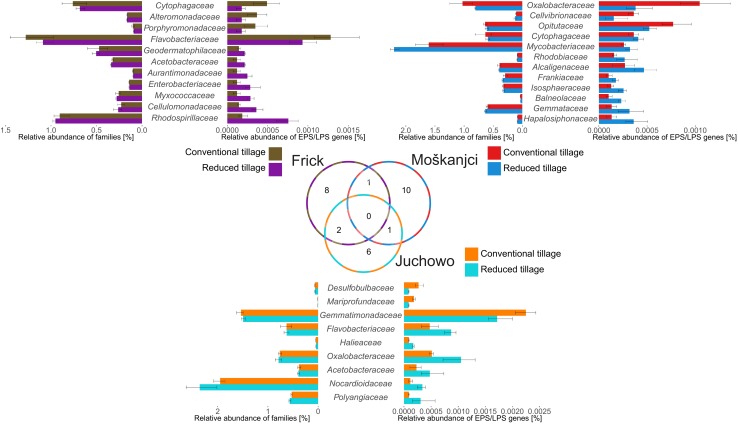
Families whose relative abundance of genes involved in the production of EPSs and LPSs was affected by tillage intensity. The Venn diagram shows the number of families that responded to tillage at each trial. The tornado plots compare the relative abundances of the responsive families with the relative abundances of their sequences related to EPS and LPS formation. The responsive families are sorted according to the log2 fold change in their relative abundances of EPS and LPS production-related sequences between conventional and reduced tillage. Error bars show standard deviations.

While no general trends in the response to tillage were identified by robust ANOVA, robust *t*-test detected that the number of reads assigned to the genes of interest differed significantly between tillage types for 11 families in Frick, for 12 families in Moškanjci, and for 9 families in Juchowo. The statistical data for the families that responded to tillage intensity can be taken from Supplementary Table S7. Comparison of those families (Figure 3) revealed that their response to tillage type was trial-dependent. No family responded to tillage treatment in all three trials, but *Acetobacteraceae* showed higher potential for EPS and LPS formation under RT compared with CT in both Frick (0.00018% under RT vs. 0.00011% under CT) and Juchowo (0.00082% under RT vs. 0.00060% under CT). Surprisingly, some bacterial families responded differently when trials were compared. *Flavobacteriaceae* had higher potential for EPS and LPS biosynthesis under CT in Frick (0.00111% under CT vs. 0.00083% under RT), and showed the opposite behavior in Juchowo (0.00079% under RT vs. 0.00039% under CT). *Cytophagaceae* also had higher potential for EPS and LPS formation under CT in Frick (0.00043% under CT vs. 0.00007% under RT), but contrasting results were observed in Moškanjci (0.00032% under RT vs. 0.00026% under CT). Similarly, *Oxalobacteracea* showed higher potential for EPS and LPS biosynthesis in Moškanjci under CT (0.00090% under CT vs. 0.00029% under RT), but in Juchowo this bacterial family had higher potential for adhesive polysaccharide production under RT (0.00096% under RT vs. 0.00042% under CT). Out of the families affected by tillage intensity at each trial, more families (63.6% in Frick, 75.0% in Moškanjci, and 66.7% in Juchowo) showed higher potential for EPS and LPS formation under RT compared with CT. Amongst the families that responded to the type of tillage, members of *Actinobacteria* (*Geodermatophilaceae* and *Cellulomonadaceae* in Frick, *Mycobacteriaceae* and *Frankiaceae* in Moskanjci, as well as *Nocardioidaceae* in Juchowo) were found, and showed higher potential for EPS and LPS formation under RT compared with CT. Interestingly, families belonging to *Actinobacteria* were also the major responding groups to the type of tillage when total bacterial diversity was assessed (see above).

## Discussion

### Link Between Soil Aggregate Stability, Tillage Intensity and Soil Texture

Preservation of soil aggregates is vital for soil sustainability in agro-ecosystems (Amezketa, 1999; Bronick and Lal, 2005). However, it is still not completely understood how soil aggregation is affected by agricultural practices such as different forms of tillage. Therefore, we investigated the effects of CT and RT on the bacterial potential to produce soil-aggregating agents such as EPSs and LPSs in agricultural trials with different soil properties. We showed that bacteria could be important drivers of aggregate formation and stabilization, but the effects of tillage intensity on soil aggregates strongly depend on site-specific properties.

The investigated sites differed in their aggregate stability, soil texture and organic carbon content. The most stable aggregates and the highest clay and organic carbon content were found in Frick, whereas least stable aggregates and the lowest clay and organic carbon content characterized the Juchowo site. Clay particles have the highest propensity compared with sand and silt particles to attract each other, form bonds and, in consequence, soil aggregates (Bronick and Lal, 2005; Weil and Brady, 2017; Totsche et al., 2018). Similarly, organic carbon is one of the most important binding agents responsible for the cementation of soil particles and aggregate formation and stabilization (Bronick and Lal, 2005). Therefore, the differences in clay and organic carbon content could largely explain the variability in the stable aggregate fraction between the three sites. However, we could also demonstrate a connection between the stable aggregate fraction and the genes involved in the biosynthesis of EPSs and LPSs. Using Cmic values as a reference, we estimated that most of the investigated genes related to the formation of these adhesive polysaccharides (83.3%) were the most abundant in Frick, and the lowest abundance values were found in Juchowo. Furthermore, the estimated absolute abundances could explain the variability in the aggregate stability between tillage intensities as well, which was impossible using clay and organic carbon content alone. Similarly to other studies (Jacobs et al., 2009; Mikha et al., 2013; Bartlova et al., 2015; Sheehy et al., 2015; Singh et al., 2016), we observed more stable soil aggregates under less intense tillage in Moškanjci. However, in Frick and Juchowo, there was no significant difference in the stable aggregate fraction under CT and RT. While soil texture did not differ significantly between tillage intensities at any of the sites, and organic carbon content was higher under less intensive tillage at all three sites, the highest number of investigated genes (66.7%) affected by tillage intensity was found in Moškanjci (compared with 25.0% in Frick and 8.3% in Juchowo). Moreover, all of the genes affected by tillage treatment in Moškanjci had higher estimated absolute abundances under RT compared with CT. These results confirm the relationship between the bacterial genes involved in EPS and LPS formation and the stable aggregate fraction.

However, the question remains why the bacterial potential to form adhesive polysaccharides was affected most in Moškanjci compared with Frick and Juchowo. This could have been caused by the longest duration of the trial in Moškanjci (continuous for 19 years from 1999 until the time of sampling) compared with Frick (16 years) and Juchowo (6 years), as it was shown before that the effects of tillage on soil properties build up over time (Stockfisch et al., 1999; Grandy et al., 2006). The fact that tillage system had a significant influence on soil pH in Moškanjci, but not the other sites, supports this theory. Alternatively, the impact of tillage system on the bacterial potential to produce adhesive polysaccharides could have been regulated by site characteristics, such as soil texture. Smaller soil particles not only can be easier aggregated together by physicochemical forces, but also by organic agents such as EPSs and LPSs, which act like a glue (Bronick and Lal, 2005; Weil and Brady, 2017; Totsche et al., 2018). Therefore, the high clay content in Frick could help stabilize the soil and also protect bacteria living inside soil aggregates from being disturbed by both CT and RT, whereas the dominance of sand in Juchowo could hinder soil aggregation and make aggregate communities equally susceptible to being disturbed by CT and RT. Overall, the difference between the level of disturbance introduced by CT and RT could be emphasized only in soils with a balanced content of small and big particles, such as in Moškanjci. This theory stresses the importance of further research on the influence of soil texture and other parameters on the response of bacteria to tillage, as this area has been so far understudied.

We based our study on the metagenomic data on EPS and LPS production as well as aggregate stability measurements as the most appropriate parameters demonstrating the long-term influence of tillage on the aggregating capabilities of bacteria. Observing the short-term effects of tillage would require measuring more dynamic parameters such as the content of bacterial polysaccharides in soil. However, while the existing methodologies of determining the polysaccharide content in this medium (Redmile-Gordon et al., 2014; Redmile-Gordon et al., 2020) do not allow for the distinction of polysaccharides from different sources, bacteria are not the only soil biota capable of producing these compounds. In fact, other organisms such as fungi or algae are also able to release polysaccharides with similar gluing properties, although these compounds are considered to be of lesser importance for soil aggregation compared with bacterial polysaccharides (Lehmann et al., 2017). Still, it would certainly be advantageous to analyze all organisms with the potential to produce adhesive polysaccharides using a single approach, especially since it would solve the issue of not being able to distinguish between different origins of polysaccharides found in soil. Unfortunately, the sequence databases are still biased toward bacteria, and therefore lacking gene sequences specific for the biosynthesis of extracellular polysaccharides. Moreover, the analysis of eukaryotes by means of short-read sequencing is difficult due to the high number of intronic sequences that these organisms possess (De Vries et al., 2015). Further work needs to be aimed at overcoming these obstacles if the full understanding of the complexities of soil aggregation is to be reached.

### Importance of EPS/LPS Genes in Agricultural Soils

While absolute abundances describe the potential significance of taxa or genes for the ecosystem functioning, the proportional importance of these groups within communities can be better explained by relative abundances. The relative abundances of the EPS/LPS genes in our study were similar to the relative abundances of functional genes analyzed in other studies, responsible e.g., for cellulose degradation (De Vries et al., 2015) or phosphorus turnover (Grafe et al., 2018), which is in line with the fact that they are not housekeeping genes. However, EPSs and LPSs increase the ecological fitness of bacteria, and therefore it could be expected that the highest relative abundance of these genes would be found amongst the most dominant taxa. EPSs and LPSs provide protection from environmental stresses and predation, mediate surface attachment, function in cell-cell and cell-plant interactions, and act as carbon reserves (Kierek and Watnick, 2003; Suresh Kumar et al., 2007; Lindhout et al., 2009). Moreover, these compounds enable bacteria to create favorable hydrological niches in soil by improving soil structure (Benard et al., 2019). In our study, the highest relative abundance of the genes related to the production of adhesive polysaccharides were harbored by *Bradyrhizobiaceae* and *Sphingomonadaceae*. Both families are common in soils, and often live in a close association with plant roots (de Souza et al., 2014; Glaeser and Kämpfer, 2014). They are also well-known producers of adhesive polysaccharides (de Souza et al., 2014; Glaeser and Kämpfer, 2014). Therefore, their high potential to form these compounds was not surprising. Furthermore, these families were amongst the most dominant families in our metagenomic datasets, and the genes involved in EPS and LPS formation were found in all of the other dominant families as well. However, the investigated genes were found not only in the most abundant families, but, in fact, in most (67.5%) of the detected families. In comparison, the genes related to the production of adhesive polysaccharides were found in 9.5 - 64.7% of bacterial families from initial ecosystems and biological soil crusts (Cania et al., 2019b). This suggests that the potential to form EPSs and LPSs is indeed an important trait for bacteria living in agricultural soils, possibly because aside from natural events, these soils are regularly disturbed by anthropogenic influences such as tillage practices.

We found very little impact of tillage intensity on both the bacterial community composition and the genes related to EPS and LPS production. Only 1.3% of all bacterial families showed a consistent response to tillage at all three sites. These responders were all families of filamentous bacteria belonging to the phylum *Actinobacteria* (Rosenberg et al., 2014), and had higher relative abundance under RT compared with CT. This suggests that bacterial filaments could be disturbed by intensive tillage in a similar manner as fungal hyphae (Beare et al., 1997; Wright et al., 1999; Borie et al., 2006; Wang et al., 2010; Kihara et al., 2012; Lu et al., 2018). These results are in line with other metagenomic analyses of agricultural soils (De Vries et al., 2015; Grafe et al., 2018) implying that bacterial communities are overall very stable, in both composition and functionality, under long-term management, and show little differences between agricultural treatments.

The genes related to the production of EPSs and LPSs maintained stable relative abundances not only under different tillage intensities, but also between the sites. Amongst the investigated genes, the only one that showed significant changes in our datasets was *wza*. This gene had higher relative abundance in Moškanjci and Juchowo compared to Frick. Previous studies showed that *wza* is an important component of the EPS synthesis pathway (Cania et al., 2019a, b). This gene encodes for an outer membrane channel transporting a variety of EPSs through the outer membrane of many different bacterial taxa (Pereira et al., 2013). Its lower relative abundance in Frick could indicate that the conditions there are more favorable for bacteria compared to Moškanjci and Juchowo, and do not require as high potential for the formation of EPSs. For example, fine-textured clayey soils offer bacteria more protection from protozoan predation than more coarse-textured silty and sandy soils (Rutherford and Juma, 1992). Similarly, soils with higher clay content tend to be better at retaining water (Amooh and Bonsu, 2015). Therefore, the protection from engulfment by protozoa and from desiccation provided by polysaccharides could possibly hold less importance in soils with high clay content, such as in Frick, compared with silty and sandy soils, such as in Moškanjci and Juchowo, respectively.

While site had little impact on the relative abundances of the investigated genes, it had an important role in shaping the composition of bacterial communities. In fact, the relative abundances of most (64.7%) detected families differed between the sites. Such strong influence of site as a factor shaping bacterial communities was expected, as soil bacteria can be affected by soil and site characteristics such as pH, climate, nutrient availability, and plant species (Fierer, 2017). These parameters varied between the investigated sites, and their impact could be seen in our metagenomic datasets, including the potential EPS and LPS producers such as the aforementioned *Bradyrhizobiaceae* and *Sphingomonadaceae*. *Bradyrhizobiaceae*, which are known for forming symbiotic associations with lupine (Reeve et al., 2013; de Souza et al., 2014), had the highest relative abundance in Juchowo, where this plant species was cultivated at the time of sampling, whereas *Sphingomonadaceae*, a bacterial family with more oligotrophic traits (Glaeser and Kämpfer, 2014), had the highest relative abundance in Moškanjci, where nutrient availability (dissolved organic carbon and nitrogen) was the lowest. The fact that these important potential producers of EPSs and LPSs differed in their relative abundance between the sites makes it surprising that site as a factor did not have a stronger influence on the relative abundance of the investigated genes. After all, the functioning of bacterial communities has been repeatedly shown to depend on their composition (Langenheder et al., 2006; Strickland et al., 2009; Reed and Martiny, 2013; Logue et al., 2016). However, some studies also found that the functional structure can be highly conserved among bacterial communities inhabiting similar environments despite their taxonomic variability (Louca et al., 2018). This is in agreement with the theory about functional redundancy, which states that a community maintains important functions even though its members may change (Allison and Martiny, 2008). Along the same lines, Fondi et al. (2016) postulated that bacterial gene pools are shaped by broad ecological niches, such as soil, sea water, inland water or host. As the bacterial potential for EPS and LPS production (i) could possibly increase the ecological fitness of bacteria living in agricultural soils, (ii) was found in most families detected in our metagenomic datasets, including all the dominant families, (iii) was maintained at a stable level by the bacterial communities at the studied sites despite different taxonomic structures, we propose that this potential is promoted mainly in agricultural soils. Whether this applies to soils in general as well as other environments requires further investigation.

### Site-Specific Response to Tillage of Potential EPS/LPS Producers

Even though the relative abundances of genes involved in the formation of EPSs and LPSs were mostly stable in our study, the aggregating capabilities of bacterial polysaccharides produced under different tillage treatments at the studied sites might have differed. In fact, even closely related bacteria can produce different types of polysaccharides (Sutherland and Thomson, 1975). At the same time, it has been shown that the slightest structural changes can lead to different properties of a polysaccharide (Suresh Kumar et al., 2007). Therefore, the aggregating capacities of polysaccharides may differ depending on which bacteria produce them. In our study, we found several bacterial families whose relative abundance of genes related to the formation of EPSs and LPSs differed under CT and RT. Even though the response of these families could be predicted to some extent, as all the responders that belonged to *Actinobacteria* had higher potential to form adhesive polysaccharides under RT compared with CT, none of the identified responsive families responded to tillage intensity at all the studied sites. This indicates that the influence of tillage on polysaccharide-producing bacteria is site-specific. Furthermore, the response of a few of the families whose potential for EPS and LPS formation was affected by tillage was inconsistent when the sites were compared. For example, the relative abundance of genes involved in the production of polysaccharides harbored by *Oxalobacteracea* was higher under CT in Moškanjci, but under RT in Juchowo. *Oxalobacteracea* are commonly found in soils, and some members of this family are employed in agriculture as plant growth-promoting agents (Baldani et al., 2014). However, little is known about the polysaccharides that they synthesize (Hiraishi et al., 1997). This underlines the importance of metagenomic studies, which enable the investigation of the community dynamics of bacterial EPS and LPS producers under natural conditions. While untargeted isolation attempts of soil-aggregating bacteria from agricultural soils yield mostly easily culturalable taxa such as *Pseudomonas* and *Bacillus* (Caesar-Tonthat et al., 2014), metagenomics could help to identify the potential key players of soil aggregation, and design more targeted isolation-based approaches. In turn, metagenomic studies, which are limited by the availability of data obtained from isolates, would benefit from additional cultivation efforts. Such complementation of different methodological approaches is especially critical, as the direct measurement of bacterial polysaccharides in soils still requires more research before they will be reliable and give more information than aggregate stability data (Redmile-Gordon et al., 2014; Costa et al., 2018).

## Conclusion

Our study shows that the bacterial potential to form EPSs and LPSs is a possible link between soil aggregate stability, tillage intensity and soil texture. Specifically, we found that improved aggregate stability was connected with increased absolute abundance of genes related to the production of adhesive polysaccharides, and that the positive effects of RT over CT were most pronounced in the soil with a balanced content of clay, silt and sand. We propose that this could be because predominantly clayey soils are stabilized by their high clay content by itself, whereas very sandy soils lack the particles that could be easily glued together by bacterial polysaccharides into stable soil aggregates. This needs to be further investigated under more controlled conditions, as field trials are characterized by many other parameters that could also influence bacterial responses. Additionally, our results show that although the potential to produce EPSs and LPSs seems to be an important trait for bacteria in agricultural soils, as they try to maintain its stable levels within their communities, tillage intensity could have an impact on the aggregating properties of bacterial polysaccharides by inducing shifts in the community of potential polysaccharide producers. As the observed effects of tillage intensity were site-specific, and were likely connected to the differences in soil texture, we propose that further research should focus on disentangling the complicities of bacterial responses to disturbances in soils with different textures.

## Data Availability Statement

The datasets generated for this study can be found in the Sequence Read Archive PRJNA555481.

## Author Contributions

BC designed the experiment, carried out the laboratory work, analyzed the data, and wrote the manuscript. GV contributed to the data analysis. MaS, RM, MK, AF, PM, and AS were responsible for the field work and chemical analyses. AF, MiS, and SS contributed to the design of the experiment. All authors edited the manuscript and approved the final draft.

## Conflict of Interest

The authors declare that the research was conducted in the absence of any commercial or financial relationships that could be construed as a potential conflict of interest.

## Abbreviations

Cmic: microbial biomass carbon
Corg: organic carbon
CT: conventional tillage
DOC: dissolved organic carbon
DON: dissolved organic nitrogen
EPS: exopolysaccharide
LPS: lipopolysaccharide
Nmic: microbial biomass nitrogen
RT: reduced tillage
SAF: stable aggregate fraction.
