# Supplementary material for: Site-Specific Conditions Change the Response of Bacterial Producers of Soil Structure-Stabilizing Agents Such as Exopolysaccharides and Lipopolysaccharides to Tillage Intensity
Source: Front Microbiol. 2020 Apr 7;11:568. doi: 10.3389/fmicb.2020.00568 (PMC7154075; doi:10.3389/fmicb.2020.00568)
Supplement: Supplementary file 1 [file Data_Sheet_1.docx]

Supplementary Material


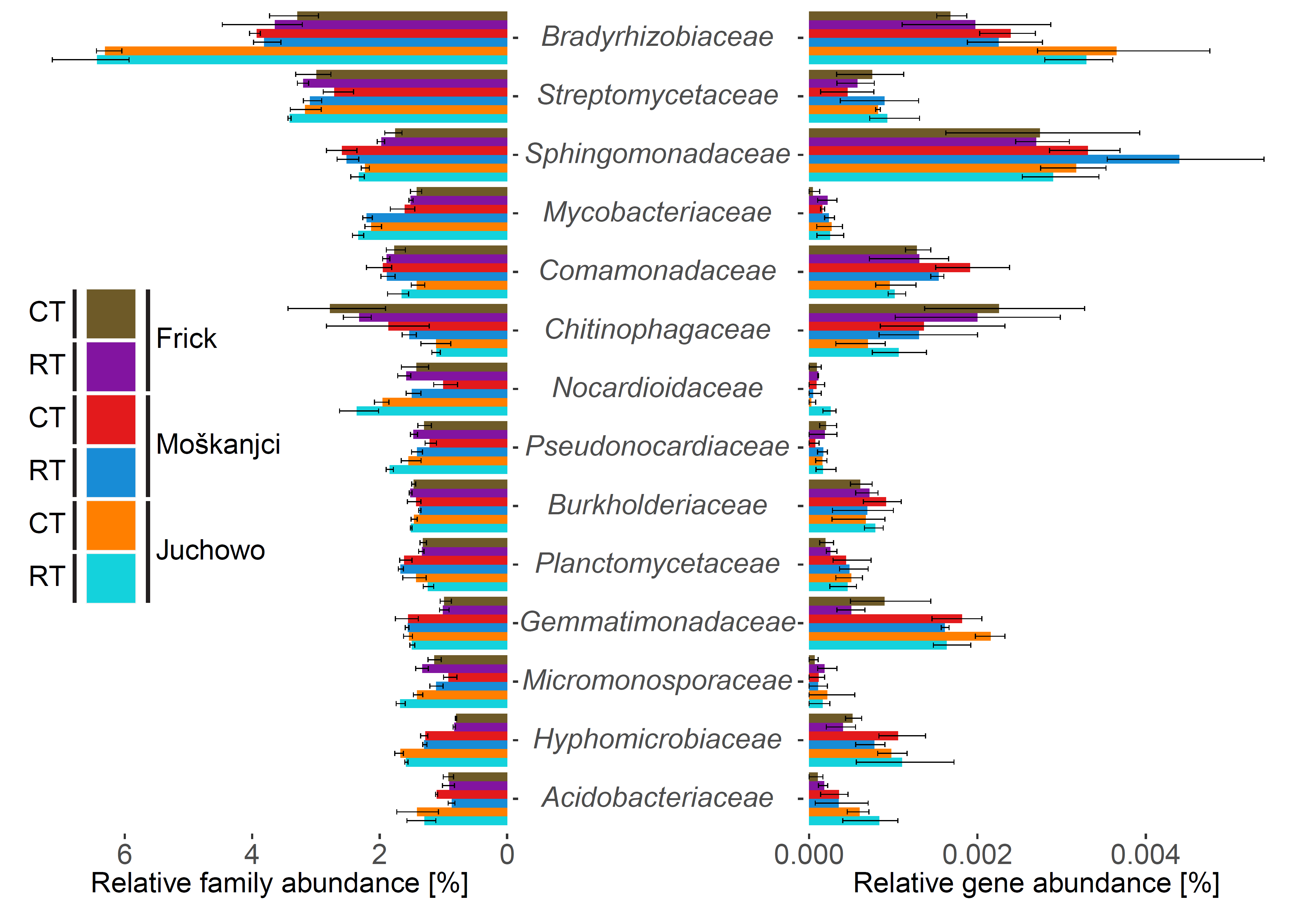


**Supplementary Figure 1.** Comparison of the relative abundances of dominant bacterial families and the relative abundances of their sequences related to EPS and LPS formation. Error bars show standard deviations.


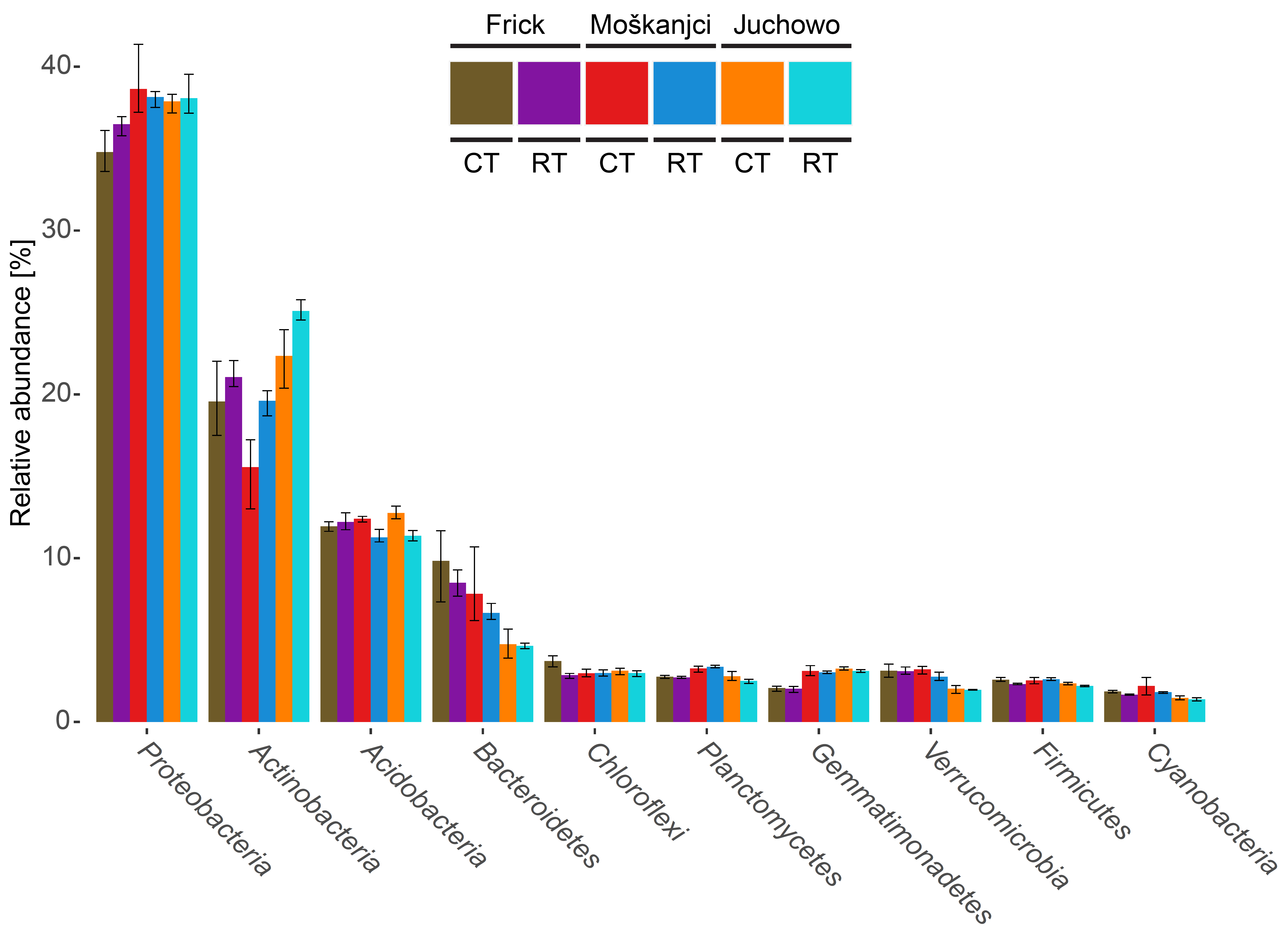


**Supplementary Figure 2.** Relative abundances of the ten most dominant phyla. Error bars show standard deviations.


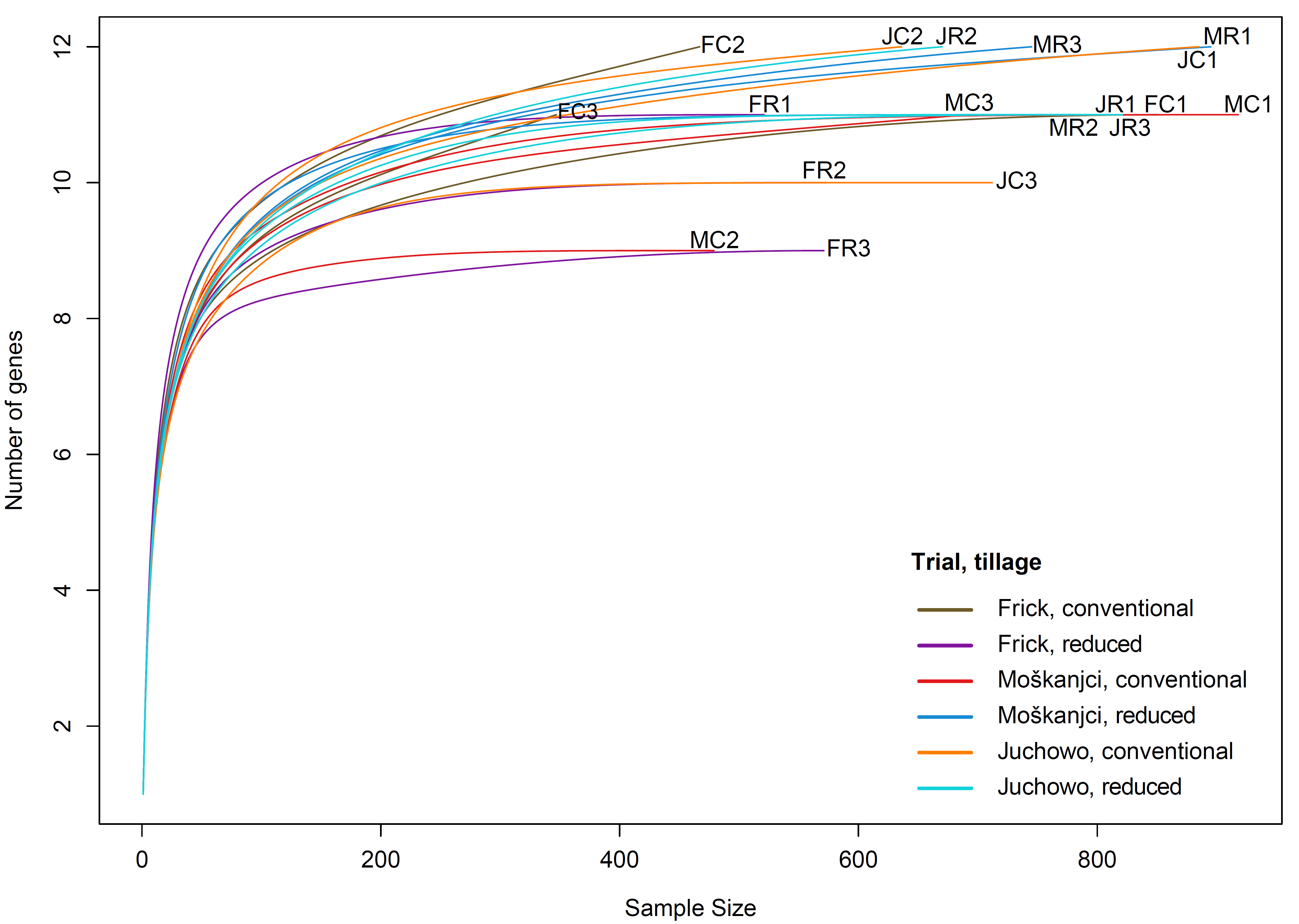


**Supplementary Figure 3.** Number of assigned genes related to the formation of EPSs and LPSs depicted as a function of sequencing depth. “F”, “M” and “J” at the beginning of the sample names stand for “Frick”, “Moškanjci” and “Juchowo”, respectively. The following letter “C” or “R” stands for conventional or reduced tillage. The number distinguishes the replicates.

**Supplementary Table 1.** Details of the raw and filtered sequencing data. “F”, “M” and “J” at the beginning of the sample names stand for “Frick”, “Moškanjci” and “Juchowo”, respectively. The following letter “C” or “R” stands for conventional or reduced tillage. The number distinguishes the replicates.

| Frick | | | | | | |
| --- | --- | --- | --- | --- | --- | --- |
| Raw data | FC1 | FC2 | FC3 | FR1 | FR2 | FR3 |
| Number of reads | 3 666 752 | 2 103 714 | 1 648 448 | 2 516 708 | 2 335 156 | 2 311 802 |
| Average length of reads [bp] | 301.00 | 301.00 | 301.00 | 301.00 | 301.00 | 301.00 |
| Filtered data |  |  |  |  |  |  |
| Number of reads | 3 666 146 | 2 102 832 | 1 647 552 | 2 516 294 | 2 334 850 | 2 311 356 |
| Average length of reads [bp] | 297.71 | 293.97 | 291.52 | 297.59 | 297.91 | 297.87 |
|  |  |  |  |  |  |  |
| Moškanjci | | | | | | |
| Raw data | MC1 | MC2 | MC3 | MR1 | MR2 | MR3 |
| Number of reads | 3 381 440 | 2 009 488 | 2 860 256 | 3 701 668 | 2 644 092 | 2 913 230 |
| Average length of reads [bp] | 301.00 | 301.00 | 301.00 | 301.00 | 301.00 | 301.00 |
| Filtered data |  |  |  |  |  |  |
| Number of reads | 3 380 688 | 2 009 194 | 2 859 498 | 3 701 194 | 2 643 614 | 2 912 892 |
| Average length of reads [bp] | 297.72 | 297.17 | 298.04 | 297.98 | 297.94 | 298.12 |
|  |  |  |  |  |  |  |
| Juchowo | | | | | | |
| Raw data | JC1 | JC2 | JC3 | JR1 | JR2 | JR3 |
| Number of reads | 3 270 470 | 2 402 428 | 2 856 936 | 3 058 810 | 2 678 352 | 3 178 412 |
| Average length of reads [bp] | 301.00 | 301.00 | 301.00 | 301.00 | 301.00 | 301.00 |
| Filtered data |  |  |  |  |  |  |
| Number of reads | 3 269 872 | 2 401 852 | 2 856 422 | 3 058 184 | 2 677 828 | 3 177 714 |
| Average length of reads [bp] | 297.81 | 297.76 | 297.91 | 297.51 | 297.64 | 297.55 |

**Supplementary Table 2.** Bacterial families whose relative abundances and potential to produce EPSs or LPSs were significantly affected by soil trial, tillage or their interaction. Significant differences were determined by a robust 2-way ANOVA (n = 3, p < 0.05).

| Factor | Families whose abundance was affected | Families whose potential to produce EPS or LPS was affected |
| --- | --- | --- |
| Trial | *Acaryochloridaceae*, *Acetobacteraceae*, *Acidiferrobacteraceae*, *Acidimicrobiaceae*, *Acidobacteriaceae*, *Acidothermaceae*, *Actinopolysporaceae*, *Actinospicaceae*, *Aerococcaceae*, *Aeromonadaceae*, *Akkermansiaceae*, *Alcaligenaceae*, *Alteromonadaceae*, *Amoebophilaceae*, *Anaerolineaceae*, *Anaeromyxobacteraceae*, *Aphanothecaceae*, *Aquificaceae*, *Archangiaceae*, *Ardenticatenaceae*, *Aurantimonadaceae*, *Bacillaceae*, *Bacteriovoracaceae*, *Bacteroidaceae*, *Balneolaceae*, *Bdellovibrionaceae*, *Beijerinckiaceae*, *Beutenbergiaceae*, *Bogoriellaceae*, *Bradyrhizobiaceae*, *Brevibacteriaceae*, *Brevinemataceae*, *Brucellaceae*, *Caldilineaceae*, *Campylobacteraceae*, *Candidatus* *Actinomarinaceae*, *Candidatus* *Brocadiaceae*, *Candidatus* *Desulfofervidaceae*, *Candidatus* *Midichloriaceae*, *Cardiobacteriaceae*, *Carnobacteriaceae*, *Catalimonadaceae*, *Catenulisporaceae*, *Cellulomonadaceae*, *Cellvibrionaceae*, *Chamaesiphonaceae*, *Chitinophagaceae*, *Chlorobiaceae*, *Chlorogloeopsidaceae*, *Chromobacteriaceae*, *Chroococcaceae*, *Chroococcidiopsidaceae*, *Chrysiogenaceae*, *Chthoniobacteraceae*, *Chthonomonadaceae*, *Clostridiaceae*, *Coleofasciculaceae*, *Colwelliaceae*, *Comamonadaceae*, *Competibacteraceae*, *Conexibacteraceae*, *Corynebacteriaceae*, *Coxiellaceae*, *Crocinitomicaceae*, *Cryomorphaceae*, *Cryptosporangiaceae*, *Cyanothecaceae*, *Cyclobacteriaceae*, *Cytophagaceae*, *Dermacoccaceae*, *Desulfobacteraceae*, *Desulfobulbaceae*, *Desulfohalobiaceae*, *Desulfomicrobiaceae*, *Desulfovibrionaceae*, *Desulfuromonadaceae*, *Dietziaceae*, *Ectothiorhodospiraceae*, *Enterobacteriaceae*, *Enterococcaceae*, *Erwiniaceae*, *Erysipelotrichaceae*, *Erythrobacteraceae*, *Eubacteriaceae*, *Ferrimonadaceae*, *Fibrobacteraceae*, *Fimbriimonadaceae*, *Flammeovirgaceae*, *Flavobacteriaceae*, *Frankiaceae*, *Fusobacteriaceae*, *Gaiellaceae*, *Gallionellaceae*, *Gemmataceae*, *Gemmatimonadaceae*, *Geobacteraceae*, *Geodermatophilaceae*, *Gomontiellaceae*, *Gordoniaceae*, *Hahellaceae*, *Halobacteriovoraceae*, *Hapalosiphonaceae*, *Helicobacteraceae*, *Herpetosiphonaceae*, *Holophagaceae*, *Hydrogenophilaceae*, *Hyellaceae*, *Hymenobacteraceae*, *Hyphomicrobiaceae*, *Idiomarinaceae*, *Ignavibacteriaceae*, *Immundisolibacteraceae*, *Intrasporangiaceae*, *Isosphaeraceae*, *Jiangellaceae*, *Jonesiaceae*, *Kangiellaceae*, *Kineosporiaceae*, *Kiritimatiellaceae*, *Kofleriaceae*, *Ktedonobacteraceae*, *Lachnospiraceae*, *Legionellaceae*, *Lentimicrobiaceae*, *Lentisphaeraceae*, *Leptolyngbyaceae*, *Leptospiraceae*, *Magnetococcaceae*, *Marinifilaceae*, *Marinilabiliaceae*, *Melioribacteraceae*, *Methylococcaceae*, *Methylocystaceae*, *Methylophilaceae*, *Methylothermaceae*, *Microbulbiferaceae*, *Micrococcaceae*, *Microcoleaceae*, *Micropepsaceae*, *Microthrixaceae*, *Moraxellaceae*, *Moritellaceae*, *Mycoplasmataceae*, *Myxococcaceae*, *Nakamurellaceae*, *Nannocystaceae*, *Nitriliruptoraceae*, *Nitrosomonadaceae*, *Nitrospinaceae*, *Nitrospiraceae*, *Nocardiaceae*, *Nocardiopsaceae*, *Nostocaceae*, *Oceanospirillaceae*, *Odoribacteraceae*, *Oleiphilaceae*, *Opitutaceae*, *Orbaceae*, *Oscillatoriaceae*, *Oscillospiraceae*, *Oxalobacteraceae*, *Pasteurellaceae*, *Patulibacteraceae*, *Pectobacteriaceae*, *Peptococcaceae*, *Peptoniphilaceae*, *Persicobacteraceae*, *Phycisphaeraceae*, *Phyllobacteriaceae*, *Piscirickettsiaceae*, *Planctomycetaceae*, *Polyangiaceae*, *Porphyromonadaceae*, *Porticoccaceae*, *Prevotellaceae*, *Prolixibacteraceae*, *Propionibacteriaceae*, *Pseudoalteromonadaceae*, *Pseudomonadaceae*, *Pseudonocardiaceae*, *Psychromonadaceae*, *Puniceicoccaceae*, *Rhizobiaceae*, *Rhodanobacteraceae*, *Rhodobiaceae*, *Rhodocyclaceae*, *Rhodospirillaceae*, *Rhodothermaceae*, *Rickettsiaceae*, *Rikenellaceae*, *Ruaniaceae*, *Rubritaleaceae*, *Rubrobacteraceae*, *Ruminococcaceae*, *Saccharospirillaceae*, *Sandaracinaceae*, *Sanguibacteraceae*, *Saprospiraceae*, *Schleiferiaceae*, *Scytonemataceae*, *Shewanellaceae*, *Solibacteraceae*, *Solirubrobacteraceae*, *Sphingobacteriaceae*, *Sphingomonadaceae*, *Spirochaetaceae*, *Spongiibacteraceae*, *Sporichthyaceae*, *Streptococcaceae*, *Streptosporangiaceae*, *Succinivibrionaceae*, *Sutterellaceae*, *Symphyonemataceae*, *Synechococcaceae*, *Synergistaceae*, *Syntrophaceae*, *Syntrophobacteraceae*, *Syntrophorhabdaceae*, *Thermaceae*, *Thermoanaerobacteraceae*, *Thermoanaerobacterales* *Family* *IV.* *Incertae* *Sedis*, *Thermomonosporaceae*, *Thermonemataceae*, *Thiotrichaceae*, *Tolypothrichaceae*, *Trueperaceae*, *Tsukamurellaceae*, *Verrucomicrobia* *subdivision* *3*, *Verrucomicrobia* *subdivision* *6*, *Verrucomicrobiaceae*, *Vibrionaceae*, *Vulgatibacteraceae*, *Wenzhouxiangellaceae*, *Williamsiaceae*, *Woeseiaceae*, *Xanthobacteraceae*, *Xenococcaceae* | *Acidobacteriaceae*, *Bdellovibrionaceae*, *Bradyrhizobiaceae*, *Comamonadaceae*, *Ectothiorhodospiraceae*, *Gemmatimonadaceae*, *Hyphomicrobiaceae*, *Micrococcaceae*, *Nocardiaceae*, *Opitutaceae*, *Sinobacteraceae* |
| Tillage | *Glycomycetaceae* | - |
| Trial + tillage | *Dermatophilaceae*, *Micromonosporaceae*, *Mycobacteriaceae*, *Nocardioidaceae* | - |
| Trial x tillage | *Acidaminococcaceae*, *Chromatiaceae*, *Halieaceae*, *Halobacteroidaceae*, *Halomonadaceae*, *Methylobacteriaceae*, *Parvularculaceae*, *Sphaerobacteraceae* | *Balneolaceae*, *Halieaceae*, *Mariprofundaceae*, *Oxalobacteraceae*, *Prolixibacteraceae*, *Rhodospirillaceae* |

**Supplementary Table 3.** Influence of soil trial, tillage and their interaction on the relative abundances of the dominant bacterial families, as determined by a robust 2-way ANOVA. Effect sizes (ω^2^) and significance levels were calculated based on triplicates (n = 3). Significance levels are represented by the amount of stars: 1 – p < 0.05, 2 – p < 0.01, 3 – p < 0.001.

| Robust ANOVA (effect sizes and significance levels) | | | | | | |
| --- | --- | --- | --- | --- | --- | --- |
| Family | Trial | | Tillage | | Trial x Tillage | |
| *Bradyrhizobiaceae* | 0.90 | *** | 0.00 | ns | 0.00 | ns |
| *Streptomycetaceae* | 0.27 | ns | 0.22 | ns | 0.00 | ns |
| *Sphingomonadaceae* | 0.77 | *** | 0.00 | ns | 0.01 | ns |
| *Mycobacteriaceae* | 0.67 | *** | 0.15 | *** | 0.07 | ns |
| *Comamonadaceae* | 0.48 | *** | 0.02 | ns | 0.02 | ns |
| *Chitinophagaceae* | 0.58 | *** | 0.01 | ns | 0.00 | ns |
| *Nocardioidaceae* | 0.68 | *** | 0.14 | *** | 0.00 | ns |
| *Pseudonocardiaceae* | 0.55 | *** | 0.24 | ns | 0.00 | ns |
| *Burkholderiaceae* | 0.30 | ns | 0.00 | ns | 0.06 | ns |
| *Planctomycetaceae* | 0.64 | *** | 0.00 | ns | 0.05 | ns |
| *Gemmatimonadaceae* | 0.88 | *** | 0.00 | ns | 0.00 | ns |
| *Micromonosporaceae* | 0.70 | *** | 0.17 | * | 0.00 | ns |
| *Hyphomicrobiaceae* | 0.98 | *** | 0.00 | ns | 0.00 | ns |
| *Acidobacteriaceae* | 0.52 | ** | 0.03 | ns | 0.00 | ns |

**Supplementary Table 4.** Influence of trial, tillage and their interaction on the relative abundances of bacterial reads assigned to genes encoding for proteins involved in EPS and LPS formation, as determined by a robust 2-way ANOVA. Effect sizes (ω^2^) and significance levels were calculated based on triplicates (n = 3). Significance levels are represented by the amount of stars: 1 – p < 0.05, 2 – p < 0.01, 3 – p < 0.001.

| Robust ANOVA (effect sizes and significance levels) | | | | | | |
| --- | --- | --- | --- | --- | --- | --- |
| Gene | Trial | | Tillage | | Trial x Tillage | |
| *wza* | 0.40 | *** | 0.00 | ns | 0.07 | ns |
| *wcaB* | 0.13 | ns | 0.00 | ns | 0.02 | ns |
| *wcaF* | 0.00 | ns | 0.06 | ns | 0.01 | ns |
| *wcaK/amsJ* | 0.03 | ns | 0.00 | ns | 0.02 | ns |
| *kpsE* | 0.07 | ns | 0.00 | ns | 0.03 | ns |
| *algE* | 0.00 | ns | 0.00 | ns | 0.00 | ns |
| *algJ* | 0.00 | ns | 0.00 | ns | 0.08 | ns |
| *sacB* | 0.03 | ns | 0.00 | ns | 0.15 | ns |
| *wzt* | 0.00 | ns | 0.00 | ns | 0.11 | ns |
| *lptC* | 0.20 | ns | 0.00 | ns | 0.01 | ns |
| *lptF* | 0.17 | ns | 0.00 | ns | 0.00 | ns |
| *lptG* | 0.27 | ns | 0.00 | ns | 0.00 | ns |

**Supplementary Table 5.** General influence of trial, tillage and their interaction, as well as site-specific influence of tillage in Frick, Moškanjci and Juchowo on the estimated absolute abundances of the EPS and LPS genes harbored by the dominant bacterial families, as determined by a robust 2-way ANOVA and a robust t-test, respectively. Corresponding effect sizes (ω^2^ and r) and significance levels were calculated based on triplicates (n = 3). Significance levels are represented by the amount of stars: 1 – p < 0.05, 2 – p < 0.01, 3 – p < 0.001.

| Robust ANOVA (effect sizes and significance levels) | | | | | | |
| --- | --- | --- | --- | --- | --- | --- |
| Gene | Trial | | Tillage | | Trial x Tillage | |
| *wza* | 0.84 | *** | 0.04 | ns | 0.03 | ns |
| *wcaB* | 0.90 | *** | 0.02 | ns | 0.00 | ns |
| *wcaF* | 0.82 | *** | 0.03 | ns | 0.00 | ns |
| *wcaK/amsJ* | 0.00 | ns | 0.00 | ns | 0.27 | ns |
| *kpsE* | 0.90 | *** | 0.03 | ns | 0.02 | ns |
| *algE* | 0.10 | ns | 0.00 | ns | 0.00 | ns |
| *algJ* | 0.52 | *** | 0.01 | ns | 0.00 | ns |
| *sacB* | 0.28 | *** | 0.01 | ns | 0.00 | ns |
| *wzt* | 0.85 | *** | 0.04 | * | 0.04 | ns |
| *lptC* | 0.75 | *** | 0.03 | ns | 0.02 | ns |
| *lptF* | 0.85 | *** | 0.03 | ns | 0.01 | ns |
| *lptG* | 0.84 | *** | 0.03 | ns | 0.01 | ns |
|  |  |  |  |  |  |  |
| Robust t-test (effect sizes and significance levels) | | | | | | |
| Gene | Frick | | Moškanjci | | Juchowo | |
| *wza* | 0.83 | *** | 0.91 | *** | 0.14 | ns |
| *wcaB* | 0.69 | ns | 0.82 | *** | 0.25 | ns |
| *wcaF* | 0.53 | ns | 0.89 | *** | 0.35 | ns |
| *wcaK/amsJ* | 0.81 | ns | 0.66 | ns | 0.38 | ns |
| *kpsE* | 0.88 | *** | 0.53 | ns | 0.37 | ns |
| *algE* | 0.18 | ns | 0.64 | ns | 0.10 | ns |
| *algJ* | 0.46 | ns | 0.77 | *** | 0.41 | ns |
| *sacB* | 0.28 | ns | 0.80 | *** | 0.94 | *** |
| *wzt* | 0.89 | *** | 0.81 | *** | 0.31 | ns |
| *lptC* | 0.65 | ns | 0.58 | ns | 0.23 | ns |
| *lptF* | 0.71 | ns | 0.85 | *** | 0.01 | ns |
| *lptG* | 0.69 | ns | 0.89 | *** | 0.00 | ns |

**Supplementary Table 6.** Influence of trial, tillage and their interaction on the relative abundances of the EPS and LPS genes harbored by the dominant bacterial families, as determined by a robust 2-way ANOVA. Effect sizes (ω^2^) and significance levels were calculated based on triplicates (n = 3). Significance levels are represented by the amount of stars: 1 – p < 0.05, 2 – p < 0.01, 3 – p < 0.001.

| Robust ANOVA (effect sizes and significance levels) | | | | | | |
| --- | --- | --- | --- | --- | --- | --- |
| Family | Trial | | Tillage | | Trial x Tillage | |
| *Bradyrhizobiaceae* | 0.55 | *** | 0.00 | ns | 0.00 | ns |
| *Streptomycetaceae* | 0.00 | ns | 0.00 | ns | 0.03 | ns |
| *Sphingomonadaceae* | 0.26 | ns | 0.00 | ns | 0.05 | ns |
| *Mycobacteriaceae* | 0.09 | ns | 0.06 | ns | 0.02 | ns |
| *Comamonadaceae* | 0.47 | *** | 0.00 | ns | 0.00 | ns |
| *Chitinophagaceae* | 0.31 | ns | 0.00 | ns | 0.00 | ns |
| *Nocardioidaceae* | 0.04 | ns | 0.10 | ns | 0.30 | ns |
| *Pseudonocardiaceae* | 0.00 | ns | 0.00 | ns | 0.00 | ns |
| *Burkholderiaceae* | 0.00 | ns | 0.00 | ns | 0.00 | ns |
| *Planctomycetaceae* | 0.29 | ns | 0.00 | ns | 0.00 | ns |
| *Gemmatimonadaceae* | 0.72 | *** | 0.08 | ns | 0.00 | ns |
| *Micromonosporaceae* | 0.00 | ns | 0.00 | ns | 0.00 | ns |
| *Hyphomicrobiaceae* | 0.39 | *** | 0.00 | ns | 0.00 | ns |
| *Acidobacteriaceae* | 0.52 | *** | 0.00 | ns | 0.00 | ns |

**Supplementary Table 7.** Influence of tillage on the overall relative abundance and the abundance of EPS and LPS genes of families whose potential for the formation of EPSs and LPSs was affected by tillage in each trial. Effect sizes (ω^2^) and significance levels were calculated based on triplicates (n = 3). Significance levels are represented by the amount of stars: 1 – p < 0.05, 2 – p < 0.01, 3 – p < 0.001.

| Robust t-test (effect sizes and significance levels) | | | | | |
| --- | --- | --- | --- | --- | --- |
| Trial | Family | Relative abundance of families | | Relative abundance of EPS/LPS genes | |
| Frick | *Cytophagaceae* | 0.50 | ns | 0.84 | *** |
|  | *Alteromonadaceae* | 0.12 | ns | 0.81 | *** |
|  | *Porphyromonadaceae* | 0.52 | ns | 0.75 | *** |
|  | *Flavobacteriaceae* | 0.59 | ns | 0.71 | *** |
|  | *Geodermatophilaceae* | 0.28 | ns | 0.92 | *** |
|  | *Acetobacteraceae* | 0.64 | ns | 1.00 | *** |
|  | *Aurantimonadaceae* | 0.26 | ns | 0.95 | *** |
|  | *Enterobacteriaceae* | 0.47 | ns | 0.89 | *** |
|  | *Myxococcaceae* | 0.64 | ns | 0.96 | *** |
|  | *Cellulomonadaceae* | 0.42 | ns | 0.94 | *** |
|  | *Rhodospirillaceae* | 0.48 | ns | 0.97 | *** |
| Moškanjci | *Oxalobacteraceae* | 0.80 | *** | 0.91 | *** |
|  | *Cellvibrionaceae* | 0.59 | ns | 0.89 | *** |
|  | *Opitutaceae* | 0.49 | ns | 0.84 | *** |
|  | *Cytophagaceae* | 0.31 | ns | 0.75 | *** |
|  | *Mycobacteriaceae* | 0.94 | *** | 0.78 | *** |
|  | *Rhodobiaceae* | 0.31 | ns | 0.73 | *** |
|  | *Alcaligenaceae* | 0.20 | ns | 0.71 | *** |
|  | *Frankiaceae* | 0.75 | *** | 0.99 | *** |
|  | *Isosphaeraceae* | 0.18 | ns | 0.86 | *** |
|  | *Balneolaceae* | 0.63 | *** | 0.97 | *** |
|  | *Gemmataceae* | 0.68 | ns | 0.83 | *** |
|  | *Hapalosiphonaceae* | 0.43 | ns | 0.87 | *** |
| Juchowo | *Desulfobulbaceae* | 0.31 | ns | 0.93 | *** |
|  | *Mariprofundaceae* | 0.31 | ns | 0.99 | *** |
|  | *Gemmatimonadaceae* | 0.43 | ns | 0.84 | *** |
|  | *Flavobacteriaceae* | 0.00 | ns | 0.88 | *** |
|  | *Halieaceae* | 0.89 | *** | 1.00 | *** |
|  | *Oxalobacteraceae* | 0.29 | ns | 0.91 | *** |
|  | *Acetobacteraceae* | 0.25 | ns | 0.69 | *** |
|  | *Nocardioidaceae* | 0.80 | ns | 0.92 | *** |
|  | *Polyangiaceae* | 0.82 | *** | 0.76 | *** |
